# Mixed Mercaptocarboxylic Acid Shells Provide Stable Dispersions of InPZnS/ZnSe/ZnS Multishell Quantum Dots in Aqueous Media

**DOI:** 10.3390/nano10091858

**Published:** 2020-09-17

**Authors:** Benjamin Heyne, Kristin Arlt, André Geßner, Alexander F. Richter, Markus Döblinger, Jochen Feldmann, Andreas Taubert, Armin Wedel

**Affiliations:** 1 Fraunhofer IAP, Geiselbergstraße 69, 14476 Potsdam, Germany; benjamin.heyne.23@gmail.com (B.H.); kristin.arlt@iap.fraunhofer.de (K.A.); andre.gessner@iap.fraunhofer.de (A.G.); 2 Photonics and Optoelectronics, Nano-Institute Munich and Department of Physics, Ludwig-Maximilians-Universität (LMU), Königinstraße 10, 80539 Munich, Germany; alexander.richter@physik.uni-muenchen.de (A.F.R.); feldmann@lmu.de (J.F.); 3 Department of Chemistry, Ludwig-Maximilians-Universität (LMU), Butenandtstraße 5-13 (E), 81377 Munich, Germany; m.doeblinger@lmu.de; 4 Institute of Chemistry, University of Potsdam, 14469 Potsdam, Germany

**Keywords:** quantum dots, QDs, cadmium-free, Cd-free, InP, InPZnS, multishell, mercaptocarboxylic acids, 3-mercaptopropionic acid, 11-mercaptoundecanoic acid, phase transfer, ligand exchange, aqueous dispersion

## Abstract

Highly luminescent indium phosphide zinc sulfide (InPZnS) quantum dots (QDs), with zinc selenide/zinc sulfide (ZnSe/ZnS) shells, were synthesized. The QDs were modified via a post-synthetic ligand exchange reaction with 3-mercaptopropionic acid (MPA) and 11-mercaptoundecanoic acid (MUA) in different MPA:MUA ratios, making this study the first investigation into the effects of mixed ligand shells on InPZnS QDs. Moreover, this article also describes an optimized method for the correlation of the QD size vs. optical absorption of the QDs. Upon ligand exchange, the QDs can be dispersed in water. Longer ligands (MUA) provide more stable dispersions than short-chain ligands. Thicker ZnSe/ZnS shells provide a better photoluminescence quantum yield (PLQY) and higher emission stability upon ligand exchange. Both the ligand exchange and the optical properties are highly reproducible between different QD batches. Before dialysis, QDs with a ZnS shell thickness of ~4.9 monolayers (ML), stabilized with a mixed MPA:MUA (mixing ratio of 1:10), showed the highest PLQY, at ~45%. After dialysis, QDs with a ZnS shell thickness of ~4.9 ML, stabilized with a mixed MPA:MUA and a ratio of 1:10 and 1:100, showed the highest PLQYs, of ~41%. The dispersions were stable up to 44 days at ambient conditions and in the dark. After 44 days, QDs with a ZnS shell thickness of ~4.9 ML, stabilized with only MUA, showed the highest PLQY, of ~34%.

## 1. Introduction

Quantum dots (QDs) have attracted tremendous attention over the last 30 years. This interest stems from their captivating size-dependent optical properties caused by the quantum confinement of the electron-hole pair [1,2,3]. QDs show high photoluminescence quantum yields (PLQYs), small full width at half maximum (FWHM), and high photostability. Absorption and emission properties can be tuned via the QD size while maintaining the same chemical composition [4,5,6,7,8]. Due to these exceptional optical properties, QDs have found application in many fields, such as displays and solar cells [9,10,11,12]. More recently, QDs have been studied for biological and diagnostic applications, such as labeling, bioimaging, and drug delivery [13,14,15,16,17,18,19]. To successfully utilize QDs in a biological and diagnostic context, however, they must be dispersed in aqueous phases for extended periods of time.

The stability of a QD dispersion in different media is determined by the composition of the ligand sphere surrounding the QD, the chemical composition and structure of the ligands, and the chemical nature of the solvent. The ligand shell stabilizes the QDs against agglomeration and protects them from QD oxidation [5]. A modification of the QD ligand shell can favor a phase transfer from organic to aqueous phases and allows for the introduction of a sufficient number of functional end groups (e.g., carboxylates, amines). These can then be further functionalized for biological and diagnostic applications.

One approach to produce QD dispersion in aqueous media is the direct synthesis in aqueous solution. However, QDs obtained via this direct route often exhibit inferior optical properties, as compared to QDs synthesized in organic solvents [20,21]. As a result, there is a certain advantage of QD synthesis in organic solvents followed by a phase transfer to the aqueous phase. Therefore, several methods for a controlled phase transfer of QDs from organic to aqueous phases exist [22,23].

One approach exploits the fact that hydrophobic ligands can be exchanged for amphiphilic ligands. For example, molecules with suitable functional groups can form a strong bond to the QD surface and thus replace hydrophobic ligands. Examples include thiols from mercapto carboxylic acids (MCAs, e.g., 3-mercaptopropionic acid MPA) or other compounds bearing thiol groups, such as penicillamine (a mercapto amino acid). In these cases, particle stabilization in the aqueous phase is achieved by the hydrophilic functional groups on the side facing the aqueous medium [24,25,26,27,28,29,30,31]. For example, Kalinowska et al. demonstrated a ligand exchange on ZnCuInS/ZnS QDs using MPA and 6-mercaptohexanoic acid. The resulting particles are stable in the aqueous phase for several months and show a high PLQY, even after phase transfer [26].

Alternatively, the ligand shell can be modified with functionalized polymers, e.g., alkylated polyethylene glycols. This procedure does not replace the existing ligand sphere on the QD. Rather, the hydrophobic residues of the polymer stabilizers interact with the hydrophobic ligand shell of the QDs and encapsulate them. Suitable polymers can be further crosslinked and an additional shell can be produced around the QDs. Such a behavior has been shown, for example, in copolymers from maleic acid anhydride and 1-octadecene or 1-tetradecene [32,33,34].

Another method for phase transfer is the silanization of the QDs. Here, an additional silica shell is grown on the QD using sol-gel processes [35,36].

Arguably the best-studied and most widely used QDs are based on cadmium. Due to the regulation provided by the Restriction of Hazardous Substances (RoHS, 2011/65/EU), the chances of Cd-based nanomaterials being approved for in vivo applications are essentially zero because of their high cytotoxicity [37,38,39]. Therefore, Cd-based QDs must be replaced with other QDs while maintaining the desired properties such as high luminescence intensity, high PLQY, and narrow FWHM. Indium phosphide (InP)-based QDs are among the current favorites, because their optical properties are quite similar to those of Cd-based QDs [8] and InP QDs show low cytotoxicity [39,40].

This article therefore describes the synthesis and properties of indium phosphide zinc sulfide (InPZnS) QDs coated with a ZnSe/ZnS shell with high dispersion stability in aqueous phases over extended periods of time. The approach is related to the studies by Allocca et al. and Mattera et al. [24,25], who used penicillamine as a stabilizer. The novelty of the current approach is twofold: (1) we provide an in-depth investigation of the effect of acetic acid treatment on the optical properties of the QDs and (2) we investigate, for the first time, the effect of ligand mixtures on QD dispersion stability in the aqueous phase. The ligand exchange, as described in this publication, presents a useful alternative to existing protocols, because MCAs are cheap, abundant, easily available in large amounts, and in many different variants. The current study focuses on MPA, 11-mercaptoundecanoic acid (MUA), and their mixtures as stabilizers for QDs in the aqueous phase, but the principle is general, highly versatile, and adaptable to other systems as well. Besides, the article also reports on an optimized method for the correlation of the QD size vs. optical absorption of the QDs. This method permits the accurate determination of InPZnS QD diameters from optical absorption data.

## 2. Materials and Methods

### 2.1. Materials

Zinc stearate (Zn(St)_2_, purum, 10–12% Zn-based), zinc oxide (ZnO, ≥99.0%), caprylic acid (CA, ≥99.0%), 1-dodecanethiol (98%), tributylphosphine (TBP, 97%), selenium (Se, ≥99.5%), sulfur (S, purum), acetone (≥99.0%), n-hexane (≥95.0%), 1-octadecene (ODE, 90%), toluene (≥99.0%), MPA (≥99%), and MUA (95%) were acquired from Sigma Aldrich (St. Louis, MO, USA). Indium acetate (In(Ac)_3_, 99.99%) and trioctylphosphine (TOP, 90%) were acquired from Alfa Aesar. Tris(trimethylsilyl)phosphine (P(TMSi)_3_, 97.5%) was obtained from Vezerf Laborsynthesen GmbH (Haverhill, MA, USA). Glacial acetic acid (HOAc, ≥99.7%) was acquired from Fisher Scientific (Hampton, VA, USA). Sodium hydroxide solution (NaOH, 1 M) was purchased from Th. Geyer GmbH & Co. KG (Renningen, Germany). Ultrapure water type II (conductivity at 25 °C is 0.055 µS/cm) was obtained from a Sartorius Arium Comfort 2 (Göttingen, Germany). All the chemicals were used without further purification unless stated otherwise.

### 2.2. Synthesis

#### 2.2.1. Precursors

Tributylphosphine selenide (TBPSe): 3.16 g (40.00 mmol) of Se were weighed into a Schlenk flask with a neck and cock, Teflon stir bar, and septum. After Se addition, the flask was slowly evacuated and filled with argon. This process was repeated three times. Then, the flask was heated with a heat gun (max. 120 °C) under a vacuum for 10 min to remove residual water. Special care was taken to ensure that the Se (melting point (mp) ~220 °C) did not melt. The flask was cooled to room temperature and flooded with argon.

Then, 11.05 mL (44.8 mmol) TBP was added under argon flow and continuous stirring. The reaction mixture was stirred for approx. 12 h at room temperature. The dissolution of the Se indicated a complete reaction. After the Se was dissolved completely, the solution was stirred for an additional 24 h at room temperature.

Trioctylphosphine sulfide (TOPS): 3.85 g (0.12 mmol) of S was weighed into a Schlenk flask with neck and cock, Teflon stir bar, and septum. After S addition, the flask was slowly evacuated and filled with argon. This process was repeated three times. The flask was heated with a heat gun (max. 95 °C) under a vacuum for 10 min to remove residual water. Special care was taken to ensure that the S did not melt (mp ~115 °C). The vessel with the reactant was cooled to room temperature and flooded with argon.

60.00 mL (134.5 mmol) trioctylphosphine (TOP) was added under argon flow and continuous stirring. The reaction mixture was stirred for approx. 12 h at room temperature. The dissolution of the S indicated a complete reaction. After the S was dissolved completely, the solution was stirred for an additional 24 h at room temperature.

Zinc octanoate (Zn(Oct)_2_): 8.14 g (0.10 mol) of zinc oxide and 28.84 g (0.20 mol) of octanoic acid were weighed into a round bottom flask with a Teflon stir bar and a reflux condenser. 200 mL of toluene was added and the reaction mixture was heated to reflux and kept for four hours. The reaction mixture was cooled to room temperature after the solution became clear and the toluene evaporated using rotary evaporation. The solid Zn(Oct)_2_ was dried overnight in a vacuum oven.

#### 2.2.2. InPZnS Multi Shell QDs

InPZnS hybrid cores were synthesized via a protocol adapted from Reference [41]. In(Ac)_3_, zinc stearate (Zn(St)_2_) or zinc octanoate (Zn(Oct)_2_), and a stir bar were added to a three-neck-flask with a septum, reflux condenser connected with the vacuum line, and a thermometer. The mixture was heated to 150 °C under vacuum and stirred for 30 min. Then, the temperature was reduced to 120 °C under argon flow. 1-dodecanethiol and HOAc were injected and the mixture was stirred for 5 min. Subsequently, the temperature was reduced to 100 °C and P(TMSi)_3_ solution (1 M in ODE) was rapidly injected into the hot reaction mixture. The mixture was stirred for 10 min., heated to 300 °C, and kept at that temperature for up to 30 min. Then, the reaction mixture was cooled to room temperature (Table 1 shows the amounts of material used for sample preparation). The reaction mixtures of particles A to D were directly used for the synthesis of the two shells onto the QDs, as described below. Since samples E to J were used for high-angle annular dark-field scanning transmission electron microscopy (HAADF-STEM) imaging, they were purified using the purification protocol described below.

To synthesize the ZnSe shell, Zn(St)_2_ was added to the reaction mixture. The mixture was heated to 100 °C and kept at that temperature until the Zn(St)_2_ was melted. TBPSe (2 M in TBP) was added, the mixture was heated to 285 °C, and kept at that temperature for 6 min. Afterwards, the reaction mixture was cooled to room temperature (Table 2).

To synthesize the ZnS shell on top of the ZnSe shell, Zn(St)_2_ was added to the reaction mixture. The mixture was heated to 100 °C and kept at that temperature until the Zn(St)_2_ melted. TOPS (2 M in TOP) was added, the mixture was heated to 250 °C, and was kept for 6 min. Afterwards, the reaction mixture was cooled to room temperature. This reaction step was adapted to generate different outer shell thicknesses (Table 3) and was performed one time for particles A, two times for particles B, three times for particles C, and five times for particles D.

For purification, 40 mL of acetone was added to the reaction mixture to remove organic impurities. Upon acetone addition, the QDs and excess ligand (stearate) precipitated. The mixture was centrifuged at 4500 rpm for 3 min and the supernatant was discarded. This procedure was repeated until the supernatant was colorless.

The precipitate was then washed with hexane extraction to remove the remaining excess ligand. The solid was mixed with 3 mL of hexane in a Falcon tube and centrifuged at 8000 rpm for 3 min. Afterwards the QDs remained in the hexane phase and the stearate ligands precipitated because of low solubility in hexane. The QD/hexane mixture was added to 40 mL of acetone and centrifuged at 8000 rpm for 3 min. The QDs precipitated and the colorless supernatant was discarded. The hexane extraction was repeated until the residue was nearly colorless.

#### 2.2.3. Ligand Exchange

**Figure 1:**
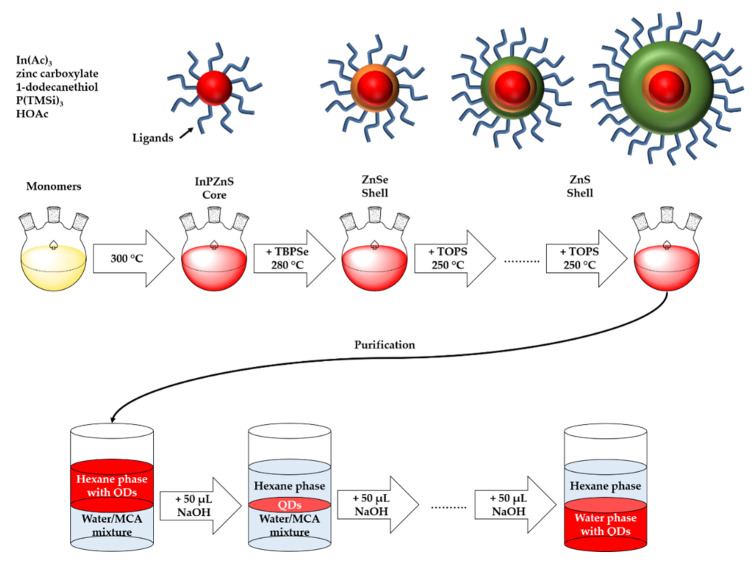
Scheme of QD synthesis and ligand exchange with MCAs.

Ligand exchange with MCAs was done via a protocol adapted from Reference [28]. 20 mg of the QDs were dispersed in 2 mL of hexane in a Falcon tube. In a second Falcon tube, 1.9 mL of water and 1.15 mmol of MPA or/and MUA (MPA/MUA ratios see Table 4) were mixed. The QD dispersion was added to the MCA-solution. Then, 50 µL of a 1 M aqueous NaOH solution was added, and the mixture was vortexed and centrifuged at 3000 rpm for 2 min. This step was repeated until all QDs were transferred into the aqueous phase (as indicated by the color of the liquid phases). Figure 1 summarizes the entire synthesis and phase transfer process.

Purification after ligand exchange was achieved as follows. The aqueous QD dispersion was removed with a syringe and passed through a polyamide syringe filter (0.2 µm) into another Falcon tube. This aqueous QD dispersion was transferred to a dialysis cassette (Fisher Scientific M_w_ cutoff ~10,000 g/mol, volume 3 mL) and dialyzed against d.i. water.

The water was changed three times (after 1, 3, and 24 h). After the final water exchange dialysis was continued for 3 h. The aqueous QD dispersion was withdrawn from the cassette using a syringe and filled into a Falcon tube through a syringe filter (PA, pore size 0.2 µm). These QDs were stored in dispersion at room temperature in the dark. For the thermogravimetric analysis, some material was dried in a drying cabinet at 50 °C under vacuum until only the QD powder remained.

### 2.3. Measurements 

UV-Vis spectra were recorded on a Perkin Elmer Lambda 19 spectrometer (Perkin Elmer, Waltham, MA, USA) from 280 to 850 nm with a resolution of 1 nm. The QDs were dispersed in toluene before and in ultrapure water after ligand exchange. All the experiments were done using quartz cuvettes with a 10 mm path length.

Photoluminescence spectra and absolute quantum yields were recorded on a Hamamatsu C9920-02 (Hamamatsu Photonics, Hamamatsu City, Japan). The QDs were dispersed in toluene before the ligand exchange and in ultrapure water after the ligand exchange. The dispersions were diluted until they showed an absorbance of 0.1 at 350 nm. Spectra were recorded from 300 to 950 nm with a resolution of <2 nm with an excitation wavelength of λ_exc_ = 350 nm (built in 150 W Xenon light source)

Thermogravimetric analysis (TGA) was performed on a Netzsch TG 209 F1 Iris thermo-microbalance (Netzsch-Gerätebau GmbH, Selb, Germany) and a TA Instruments Q500 (TA Instruments, Inc., Newcastle, DE, USA) from 25 °C to 550 °C at a heating rate of 10 K/min under nitrogen flow in an aluminum crucible. The QD samples were prepared by placing a drop of the QD in aqueous dispersion in the crucible and drying at 50 °C under a vacuum prior to the measurements.

Zeta potential was determined with a Zetasizer Nano ZS (Malvern Instruments, Malvern, UK) using a disposable capillary cell (DTS1070) and an equilibration time of 20 s. For all the measurements, 10 µL of the aqueous QD dispersion, obtained after dialysis, were diluted by a factor of 10 and directly measured afterwards.

Transmission electron microscopy (TEM) images were obtained on a JEOL JEM-1400 Plus (Jeol GmbH, Freising, Germany) operated at 120 kV. The QD samples were dispersed in toluene to a concentration of 0.01 wt%. TEM specimens were prepared by depositing a drop of the dispersion on carbon-coated copper grids and were dried in the air.

High-angle annular dark-field scanning transmission electron microscopy (HAADF-STEM) images were acquired using an FEI Titan Themis microscope operating at 300 kV. Specimens were prepared by depositing a drop of the QD dispersions on a carbon-coated copper grid and were dried in the air.

Powder X-ray diffraction (XRD) measurements were done on a Siemens D5000 equipped with a closed Eulerian cradle (Bruker-AXS) (Bruker, Billerica, USA)in reflection geometry using Ni-filtered Cu K_α_ radiation (λ = 1.5418 Å). Range was 20 to 90° 2θ and step size was 0.01°. Specimens were prepared by depositing the dry powders on Kapton foil.

Chemical composition of the powders was determined via inductively-coupled plasma optical emission spectroscopy (ICP OES, Perkin-Elmer Optima 2100 DV). (Perkin Elmer, Waltham, MA, USA) 

Elemental analysis was done on a Thermo Fisher FlashEA 1112 CHNS/O automatic elemental analyzer with two auto samplers MAS200R and a Porapack PQS column. (Thermo Fisher Scientific, Waltham, MA, USA)

### 2.4. Calculation of Core Sizes and Amounts of Shell Precursor

The absorption size dependencies for the Cd-based [42] and InP-based QDs from optical absorption and XRD data have been established [43]. In the current study, we used an extended approach, which also took into account TEM data [3,43,44]. The QD core size was calculated by assuming a spherical QD shape and the resulting shell precursor quantities were used for the syntheses of the QDs in this publication.

**Figure 2:**
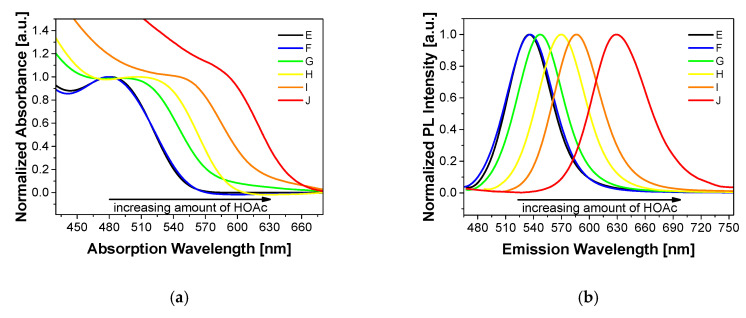
Absorption (**a**) and emission spectra (**b**) of E, F, G, H, I and J (λ_exc_ = 350 nm). Emission spectra were normalized at the emission peak wavelength.

However, TEM images showed that the QDs were smaller than those calculated from XRD and emission spectra. Therefore, six QD core samples of different absorption wavelengths, and thus core sizes, were measured by HAADF/STEM to modify the calculation of the core size via the first exciton absorption maxima. Figure 2 shows the absorption and emission spectra of particles E to J.

**Figure A1:**
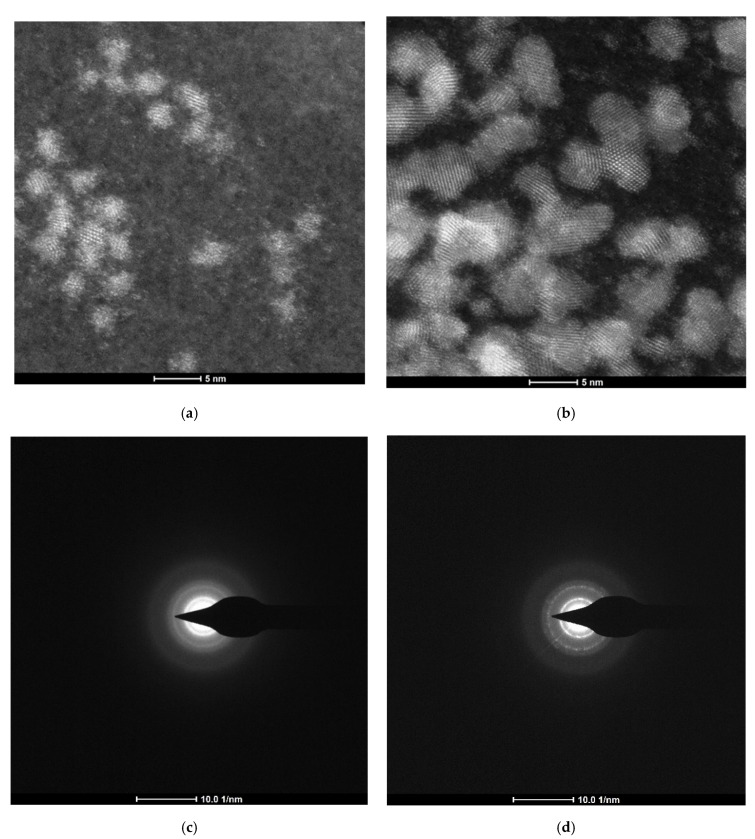
HAADF/STEM images of samples E (**a**) and J (**b**). Electron diffraction patterns of samples E (**c**) and J (**d**).

For the InPZnS hybrid core system used here, an optimized method for the calculation of the QD size vs. optical absorption correlation was developed. For this purpose, cores with different sizes were evaluated using HAADF-STEM images. Figure A1a,b (Appendix A) shows representative HAADF/STEM images of samples E and J. Analysis of the electron diffraction pattern (Figure A1c,d) yields interplanar spacings of 3.3 Å, 2.0 Å, and 1.7 Å, which corresponds to the (111), (220), and (222) or (311) planes of the InP zinc blende structure in sample E. In the case of sample J, the values for d(hkl) varied within 3.1–3.4 Å (111), 1.9–2.0 Å (220), and 1.6–1.7 Å (222) or (311). We attributed this ambiguity to the enhanced presence of ZnS in the larger QDs (see Table A1, Table A2 and Table A3, Appendix B, Appendix C and Appendix D) [45,46].

**Figure 3:**
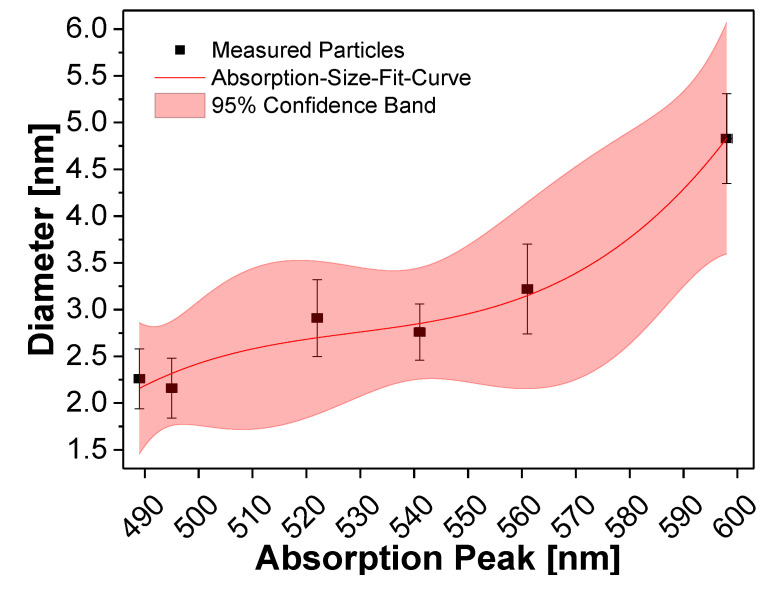
Correlation between the size and the first exciton absorption peak of the InPZnS hybrid cores E to J (black squares from left to right).

The true particle diameters were determined using GIMP (www.gimp.org) and ImageJ (www.imagej.nih.gov), assuming a spherical QD shape. The QDs were identified by hand using GIMP and the surface areas were calculated by ImageJ for each QD marked. Furthermore, particle sizes were also determined from the corresponding maxima of first exciton absorption. The first exciton absorption peak of the QDs was determined from the minimum of the second derivative of the absorption curve [47]. Combining the sizes obtained from HAADF-STEM images and exciton peaks, a calibration curve was obtained, as shown in Figure 3 (detailed data are shown in Table A4, Appendix E).

Equation (1) shows how the size of the InP based cores can be heuristically related to the absorption peak (red line in Figure 3). Using the lattice parameters of (bulk) zinc selenide (ZnSe) and zinc sulfide (ZnS), the thickness of one ZnS or ZnSe monolayer (ML) can be calculated. One ZnSe ML is 0.33 nm and one ZnS ML is 0.31 nm thick [48].

(1) $$d_{core}=4.71*10^{-6}*{\lambda_{abs}}^{3}-7.47*10^{-3}*{\lambda_{abs}}^{2}+3.96*\lambda_{abs}-697.85.\;$$

Equation (1): calculation of the size of QD cores using the first exciton absorption peak.

Assuming a 100% conversion of the starting materials, the amount of precursor needed for ZnS/ZnSe monolayer formation on the InPZnS QD core could be calculated. With the additional assumption of a uniform ML growth, the theoretical shell thicknesses can be calculated and thus the total size of the QDs can be determined. For all the QDs synthesized, the shell thicknesses were calculated from the precursor quantities used.

## 3. Results

### 3.1. QD Synthesis and Characterization

Table 5 shows the composition and basic characterization data of the different QDs. The shell thicknesses in the numbers of monolayers were calculated using the position of the first exciton absorption peak and the amount of TBPSe and TOPS, assuming 100% conversion (Section 2.4).

**Figure 4:**
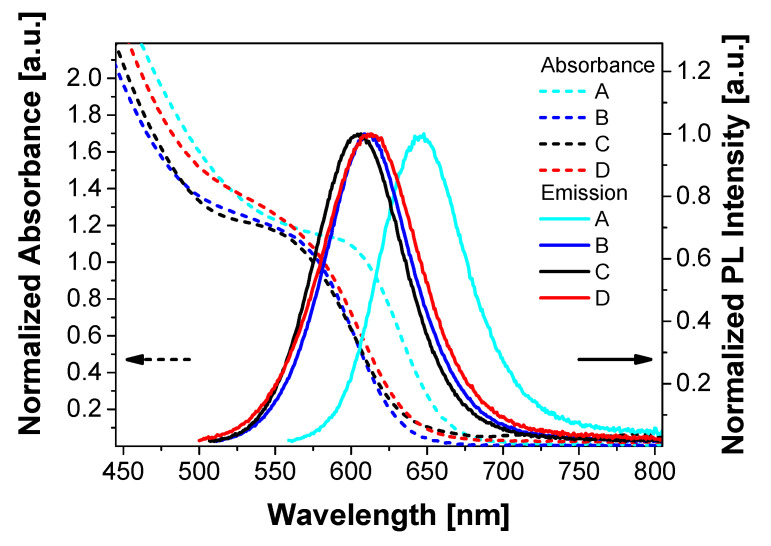
Absorption (left) and emission spectra (right) of the A, B, C, and D QDs redispersed in toluene after purification (λ_exc_ = 350 nm). Emission spectra were normalized at the peak emission wavelength.

Figure 4 shows the absorption and emission spectra of QD materials A, B, C, and D redispersed in toluene after purification. The absorption spectra show shoulders (first exciton absorption peak) at 610, 579, 575, and 582 nm, respectively. Between B, C, and D both the absorption and emission peaks show a shift of up to 7 nm from B to D, correlating with the particle size and shell thicknesses. B, C, and D also show Stokes shifts of 30 to 31 nm. A Stokes shift of 38 nm from QD material A can be observed. The PLQYs decrease with increasing emission wavelength. Samples B, C, and D show PLQYs of 60%, 52%, and 42%, respectively. In contrast, the PLQY of A is only 31%. The FWHM values show a small increase from A to D. This correlates with increasing batch size and presumably with a slight increase in size distribution (less homogeneous nucleation) as the reaction volumes increase from samples A to D.

**Figure 5:**
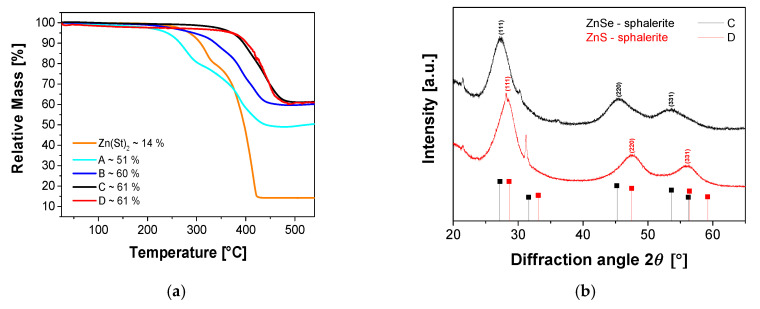
TGA curves of A to D (**a**) and XRD patterns of C and D (**b**) are shown. The vertical lines show the reflections of the reference structures of ZnSe zinc blende [49] and ZnS zinc blende [50]. The sharp reflections at 30.2 and 31.24° 2θ are from the sample holder.

TGA data of particles A to D, as well as Zn(St)_2_, are shown in Figure 5a. TGA data of particles A and B show two weight losses, similar to Zn(St)_2_. In sample A, the onsets of these weight losses are observed at lower temperatures (~210 and ~295 °C), as compared to Zn(St)_2_, while sample B (~230 and 330 °C) shows onsets in approximately the same temperature range as Zn(St)_2_ at ~235 and ~330 °C. TGA data of particles C and D are different in that they only show one weight loss with an onset at ~350 °C. At 550 °C the residual mass of A is 51% indicating an organic fraction (i.e., ligand and free ligand that could not be removed during washing) of approx. 49%. Particles B, C, and D have a residual mass between 60% and 61% at 550 °C, indicating an organic fraction of approx. 39% to 40%.

Figure 5b shows the corresponding XRD data of particles C and D. The reflections are broad, which indicates the presence of nanocrystals and defects. The patterns of the C QDs show reflections at 27.4, 45.5, and 53.0° 2θ. These are the (111), (220), and (331) reflections of the ZnSe zinc blende structure. The reflections observed in the patterns of the D QDs can be assigned to the ZnS zinc blende structure and are observed at 28.2, 47.5, and 56.1° 2θ. These positions correspond to the (111), (220), and (331) reflections.

**Figure A2:**
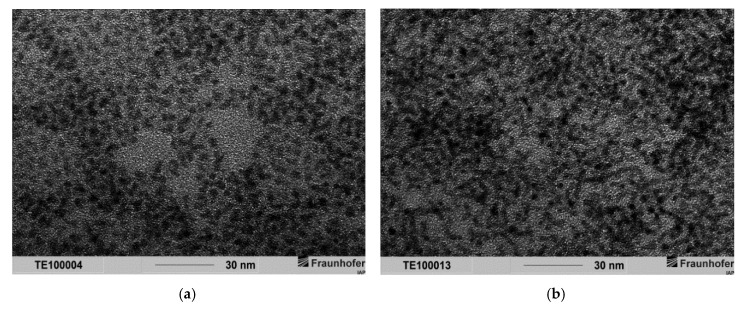
TEM images of samples C (**a**) and D (**b**).

The nanometer size of the particles is further confirmed by transmission electron microscopy (TEM). Figure A2 (Appendix F) shows representative TEM images of the same samples, C and D. TEM indicates that the particles have roughly spherical shapes and diameters of <10 nm.

Table 6 shows the ICP OES data obtained for C and D. The atomic contents of the elements In, P, Zn, S, and Se were normalized to the atomic content of In. C and D have the same P content in relation to In. In contrast, QDs C have double the Se content, indicating the presence of the first shell, consisting of ZnSe, which may be slightly thicker than in the QDs D. However, the amount of Zn and S was significantly higher in D than in C. Overall, ICP OES showed a ca. 3.45 times more than ZnS was present in sample D, which is consistent with the precursor concentrations used in the synthesis.

### 3.2. Ligand Exchange

#### 3.2.1. Phase Transfer of QDs with Different Shell Thicknesses

**Figure 6:**
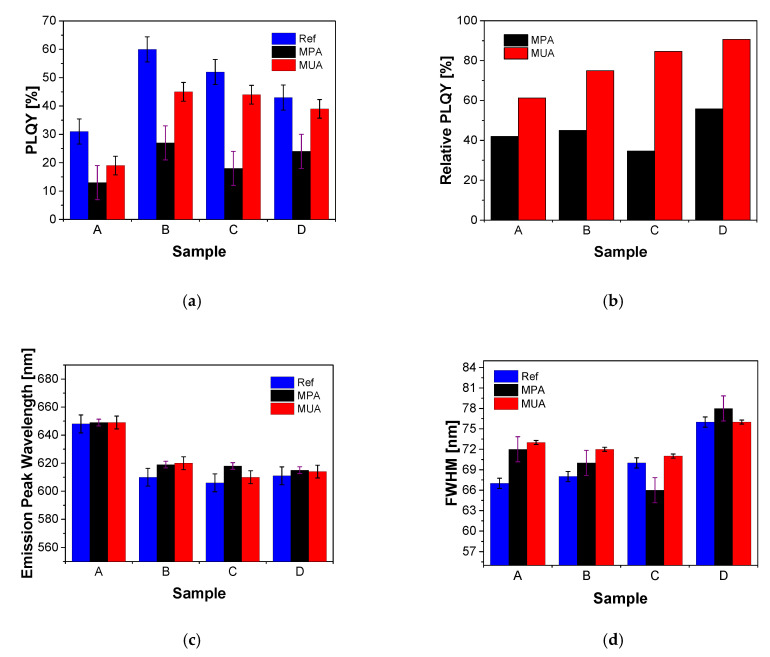
Absolute PLQY (**a**), relative PLQY (**b**), emission peak wavelengths (**c**), and FWHM (**d**) of QDs with different shell configurations before (blue) and after ligand exchange with MPA (black) and MUA (red). Relative PLQY (**b**) is the ratio between absolute PLQY of QDs dispersed in water and of reference QDs (optical properties of samples A to D before ligand exchanges dispersed in toluene) as a function of the reference samples.

The following section describes the influence of the shell thicknesses on stability during ligand exchange. Figure 6a shows the absolute PLQYs, Figure 6b shows the ratios of the PLQYs of the QDs dispersed in water vs. a reference sample in toluene, Figure 6c shows the emission peak wavelengths, and Figure 6d shows the FWHMs of samples A, B, C and D before and after ligand exchange with MPA and MUA (data from Table A5, Appendix G).

Upon ligand exchange, a PLQY loss was observed for each shell configuration. The QDs coated with MPA show higher PLQY losses than QDs coated with MUA. The highest PLQY losses of 33% and 34%, respectively, were observed for samples B and C after ligand exchange with MPA. D QDs coated with MPA showed a smaller reduction and A had the smallest reduction of the PLQY with 19% and 13%, respectively. The MUA coated QDs B and C showed the highest absolute PLQYs after ligand exchange (45% and 44%). The MUA coated sample D had an absolute PLQY of 39%. The PLQY losses of 15%, 8%, and 4% for B, C and D, stabilized with MUA, were significantly lower compared to QDs coated with MPA (Figure 6a). Overall, when considering the individual PLQYs normalized to the reference PLQY, the particles coated with MUA showed that thicker shells lead to less pronounced PLQY losses (Figure 6b).

In addition, a red shift of the emission wavelengths of 1, 11, 12, and 4 nm could be observed after the ligand exchange with MPA and a corresponding shift of 1, 12, 4, and 3 nm was observed after the ligand exchange with MUA for samples A, B, C and D, respectively (Figure 6c). In contrast to the PLQY and emission wavelength, the FWHM did not change in a systematic way. All the FWHM values varied between ±5 nm, as compared to the reference FWHM (Figure 6d). All FWHM changes were quite small.

**Figure A3:**
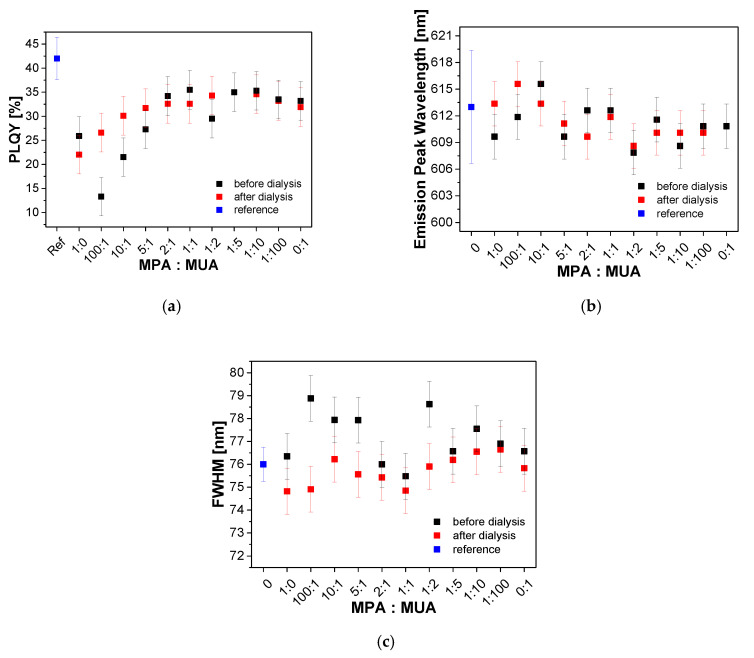
Absolute PLQY (**a**), emission peak wavelengths (**b**) and FWHM values (**c**) of D QDs with different molar ratios of MPA and MUA used for the ligand exchange are shown before and after dialysis.

QDs C showed the lowest quantum efficiency losses, smallest shift of emission peak wavelength, smallest change of FWHM, and the highest absolute PLQY after ligand exchange with MUA. QDs C were therefore chosen for further investigation of the ligand exchange reaction with MPA:MUA mixtures. For comparison purposes, the results of the D QDs coated with mixtures of MPA and MUA are shown in Figure A3 (Appendix H).

#### 3.2.2. Phase Transfer of QDs Using Mixtures of MPA and MUA

**Figure 7:**
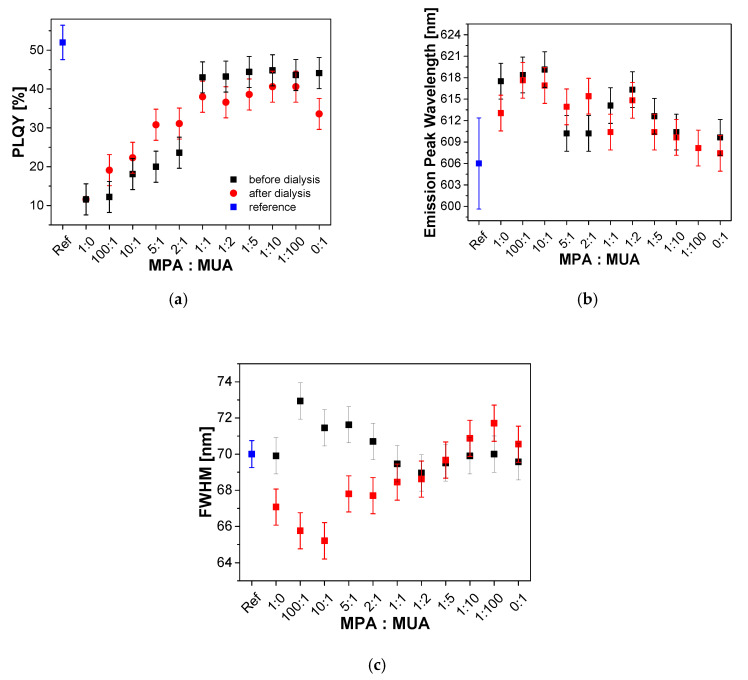
Absolute PLQY (**a**), emission peak wavelengths (**b**) and FWHM values (**c**) of C QDs with different molar ratios of MPA and MUA used for the ligand exchange are shown before and after dialysis.

Figure 7 shows the optical properties of C QDs before (data from Table 5) and after ligand exchange with nine different MPA/MUA ratios (data from Table A6 and Table A7, Appendix I and Appendix J). The errors were estimated based on an investigation of three samples, each for the ligand exchange with only MPA, MPA:MUA = 1:1, and only MUA. The largest error of approx. 4% for the PLQY was determined for the samples treated with MUA only. The errors of the emission peak wavelengths and FWHM values were approx. 2.5 and 1 nm, respectively. A clear trend of increasing PLQY from, 12% to 43%, before dialysis, and from 12% to 38% after dialysis, was observed with an increasing MUA content between a MPA:MUA ratio of 1:0 and 1:1. A constant PLQY could be seen between a MPA:MUA ratio of 1:1 and 0:1.

A comparison of the data suggests two regimes before and after dialysis. First, the PLQY of the samples after dialysis seemed to be slightly higher than before dialysis up to a MPA:MUA ratio of 2:1. Starting at a MPA:MUA ratio of 1:1, this effect was reversed. Here, the samples after dialysis showed a slightly lower PLQY compared to before dialysis (approx. up to 10%). Second, the PLQY did not vary significantly with a MPA:MUA ratio < 1:1. The only outlier seemed to be the sample which was treated with MUA only, where the PLQY dropped significantly by an absolute value of ~10%, from 44% to 34% after dialysis, as compared to a rather slight decrease by ~6% observed for the other samples. However, although the data points seem to indicate a clear trend for both regimes, this has to be proven with a more exact measurement. As we assumed a rather large error of 4% in the PLQY based on the reproducibility experiment, the error bars slightly overlapped and a final conclusion could not be made at this point. In addition, a trend could be observed in the peak emission wavelength for the different ratios of MPA to MUA. The lower the MPA:MUA ratio, the smaller the shift of the peak emission wavelength becomes. For the FWHM, a less systematic change in the region of 69 nm ± 4 nm was observed.

**Figure A4:**
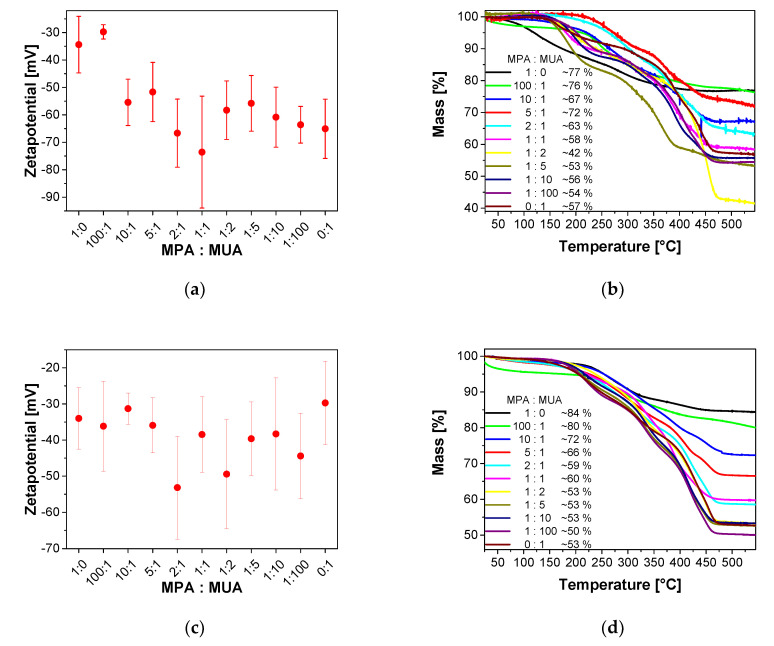
Zetapotentials (**a**,**c**) and TGA patterns (**b**,**d**) of samples C (**a**,**b**) and D (**c**,**d**) coated with different molar ratios of MPA and MUA ligands after dialysis.

Zeta potential measurements of the aqueous QD dispersions just described (Figure A4a,c) showed that the zeta potential was always below −30 mV. As a result, the dispersions were stable [51].

TGA data of same materials are shown in Figure A4b,d. A rough trend can be identified as the data show an increasing residual mass with an increasing amount of MPA ligands, i.e., less organic material, which proves that ligands are substituted during the process described above.

#### 3.2.3. Long Term Stability Test

**Figure 8:**
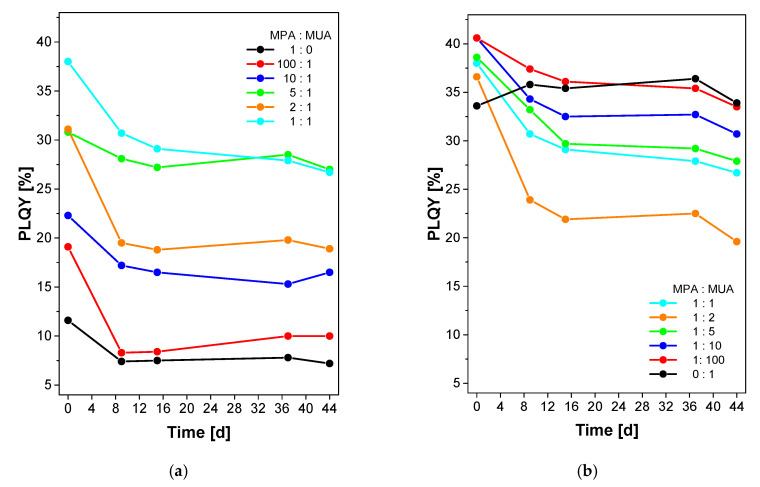
Absolute PLQY measurements of dispersions re-measured up to 44 days after preparation showing the long term stability of the C QDs coated with different MPA:MUA ratios after dialysis and storage in air and in the dark. (**a**) MPA:MUA ratio from 1:0 to 1:11; (**b**) MPA: MUA ratio from 1:1 to 0:1. Error bars are omitted for clarity, see Figure A5 (Appendix L) for identical figures with error bars added. Time d is days.

After dialysis, the QD samples were stored in Falcon tubes under air and in the dark. The optical properties of these samples were recorded after 9, 15, 37, and 44 days, respectively. Figure 8 shows that the PLQY generally decreases within the first nine days. The only exception is the sample with only MUA, which shows a stable PLQY over the whole time period. The highest PLQY reduction was recorded for particles coated with MPA:MUA at 100:1, 2:1, and 1:2 with losses of 11%, 12%, and 13%, respectively. After the first PLQY drop, all PLQYs remain roughly constant with further losses smaller than 4%. No PLQY drops below ~50% of the initial PLQY after 44 days.

## 4. Discussion

As stated in the introduction, the quest for new, Cd-free QDs poses various challenges, starting (1) with the synthesis of suitable materials on to (2) the search for particles with equal or better optical properties than those known from Cd-based QDs. A further challenge (3) arises when facing the problem of QD dispersion in aqueous (rather than organic) media. This article introduced a series of improved and highly promising QDs that (1) are entirely Cd-free, (2) show very good PLQY stability in spite of a ligand exchange, and (3) can be dispersed in water with the dispersions remaining stable for over a month without a significant loss in optical performance.

The synthesis is, although a multistep process, rather straightforward (Figure 1) and produces pure and uniform products (Figure 5). In particular, there is no need to isolate or purify individual intermediate products. Rather, the process works by simple adjustment of precursor ratios and reaction temperature (changes) producing quite uniform QDs. Moreover, the synthetic process can be adapted to directly generate different shell thicknesses. This greatly simplifies the work flow compared to earlier approaches [44,52,53].

The ligand exchange introduced here (Figure 1) is quite straightforward as well. It is based on simple ligands that are (1) commercially available in large amounts, cheap, and available in many different variants, and (2) can in principle be obtained from renewable resources such as fats and oils. Moreover, the ligand exchange is quite effective and the resulting dispersions are stable for up to approximately 6 weeks without loss of stability, flocculation, aggregation, or particle degradation (Figure 8).

The two-phase ligand exchange is used in many protocols for CdSe [51,54,55,56,57,58], but so far there are only a few systematic studies on InP-based QD systems [28,59,60]. For example, Tamang et al. found a decrease in PLQY with an increasing chain length of the MCA stabilizers [28]. In contrast, our MUA modified QDs show higher PLQYs compared to MPA modified QDs, which shows a similar trend compared to Cd based QDs [61]. This could indicate that the ligand passivation is more efficient with MUA than with MPA. Presumably the thicker ligand shell provided by the MUA provide better protection against the aqueous phase. Moreover, the optical properties can be influenced by the shell thickness (Table 5). The modification of the particle surface with the MCA ligands does alter the optical properties, but they remain such that the resulting particles are interesting for applications in aqueous environments.

Finally, this article also provides an updated and extended model approach for the correlation of the QD size with the optical absorption properties. This is a potentially very helpful tool for more accurate QD size determinations from optical absorption data.

## 5. Conclusions

This article describes the synthesis and optical properties of a series of core/shell/shell QDs with complex compositions and their transfer into the aqueous phase via a ligand exchange reaction with high reproducibility. The key novelty of the study is the proof that mixtures of cheap and abundant ligands are efficient stabilizers for luminescent QDs in aqueous media; this study is in fact the first investigation showing that ligand mixtures are powerful tools for improving the dispersion stability of QDs. Careful selection of the stabilizing ligands and their mixtures enable the preservation of the optical properties of the QDs and the production of highly stable QD dispersions. Before dialysis, PLQYs of up to 45% ± 4% in aqueous dispersion were achieved. Depending on the exact composition of the ligand shell and even after dialysis particles still showed PLQYs of up to 44% ± 4% in aqueous dispersion. All QDs showed a good long term stability in air and in the dark. Particles stabilized with only MUA showed no drop in PLQY over 44 days of storage. Overall, the QDs are thus interesting for applications in aqueous environments and further efforts need to focus on improving QD quality and additional surface functionalization (e.g., with antibodies or aptamers).

## Figures and Tables

**Table 1:** Amounts of In(Ac)_3_, Zn(St)_2_, and Zn(Oct)_2_ and volumes of 1-dodecanethiol, HOAc, and P(TMSi)_3_ (1 M in ODE) of different QD-samples used for synthesis of the InPZnS hybrid cores.

Sample Name	In(Ac)_3_ [g, (mmol)]	Zn(St)_2_ [g, (mmol)]	Zn(Oct)_2_ [g, (mmol)]	1-Dodecanethiol [mL, (mmol)]	HOAc [mL, (mmol)]	P(TMSi)_3_ [mL, (mmol)]
A	0.58 (2.00)	2.53 (4.00)	-	0.24 (1.00)	0.30 (5.25)	2.00 (2.00)
B	0.73 (2.50)	3.16 (5.00)	-	0.30 (1.25)	0.30 (5.25)	2.50 (2.50)
C	0.73 (2.50)	3.16 (5.00)	-	0.30 (1.25)	0.48 (8.39)	2.50 (2.50)
D	1.17 (4.00)	5.60 (8.00)	-	0.48 (2.00)	0.30 (5.25)	3.00 (3.00)
E	0.29 (1.00)	-	0.70 (2.00)	0.12 (0.50)	0.00 (0.00)	1.00 (1.00)
F	0.29 (1.00)	-	0.70 (2.00)	0.12 (0.50)	0.02 (0.35)	1.00 (1.00)
G	0.29 (1.00)	-	0.70 (2.00)	0.12 (0.50)	0.03 (0.52)	1.00 (1.00)
H	0.29 (1.00)	-	0.70 (2.00)	0.12 (0.50)	0.05 (0.87)	1.00 (1.00)
I	0.29 (1.00)	-	0.70 (2.00)	0.12 (0.50)	0.09 (1.57)	1.00 (1.00)
J	0.29 (1.00)	-	0.70 (2.00)	0.12 (0.50)	0.30 (5.25)	1.00 (1.00)

**Table 2:** Amounts of TBPSe and Zn(St)_2_ added for the formation of the ZnSe-shell onto the InPZnS QDs.

Sample Name	TBPSe [mL, (mmol)]	Zn(St)_2_ [g, (mmol)]
A	0.10 (0.20)	0.00 (0.00)
B	0.28 (0.56)	3.00 (4.74)
C	0.42 (0.84)	4.00 (6.33)
D	0.58 (1.06)	3.00 (4.74)

**Table 3:** Amounts of TOPS and Zn(St)_2_ used for the synthesis of the different ZnS shells on the InPZnS/ZnSe QDs.

	Step 1		Step 2		Step 3		Step 4		Step 5	
Sample	TOPS [mL, (mmol)]	Zn(St)_2_ [g, (mmol)]	TOPS [mL, (mmol)]	Zn(St)_2_ [g, (mmol)]	TOPS [mL, (mmol)]	Zn(St)_2_ [g, (mmol)]	TOPS [mL, (mmol)]	Zn(St)_2_ [g, (mmol)]	TOPS [mL, (mmol)]	Zn(St)_2_ [g, (mmol)]
A	0.31 (0.62)	0.00 (0.00)	-	-	-	-	-	-	-	-
B	1.02 (2.04)	0.00 (0.00)	1.34 (2.68)	0.00 (0.00)	-	-	-	-	-	-
C	1.69 (3.38)	0.00 (0.00)	2.41 (4.81)	6.00 (9.49)	5.28 (10.56)	0.00 (0.00)	-	-	-	-
D	2.20 (4.40)	6.00 (9.49)	3.98 (7.96)	3.02 (4.77)	3.98 (7.96)	16.30 (25.78)	15.42 (30.84)	0.00 (0.00)	34.14 (68.28)	16.30 (25.78)

**Table 4:** Volumes of MPA and amounts of MUA, which were added during ligand exchange.

MPA/MUA Molar Ratio	MPA [µL, (mmol)]	MUA [mg, (mmol)]
1:0	100 (1.15)	0.0 (0.00)
100:1	99 (1.14)	2.5 (0.01)
10:1	91 (1.05)	22.8 (0.10)
5:1	83 (0.96)	41.8 (0.19)
2:1	67 (0.77)	83.5 (0.38)
1:1	50 (0.57)	125.3 (0.57)
1:2	33 (0.38)	167.1 (0.77)
1:5	17 (0.19)	208.8 (0.96)
1:10	9 (0.10)	227.8 (1.05)
1:100	1 (0.01)	248.1 (1.14)
0:1	0 (0.00)	250.6 (1.15)

**Table 5:** Shell thickness of monolayers (ML), total diameters, and optical properties of the QDs. PLQY is the photoluminescence quantum yield and FWHM is full width at half the maximum of the emission peak.

QD Sample Name	ZnSe Shell Thickness [ML]	ZnS Shell Thickness [ML]	Total Diameter [nm]	First Exciton Absorption Wavelength [nm]	Emission Peak Wavelength [nm]	PLQY [%]	FWHM [nm]
A	0.2 (+0.0; −0.2)	0.6 (+1.0; −0.2)	5.8 (+0.9; −1.8)	610	648	31	67
B	0.3 (±0.1)	1.8 (±0.6)	4.7 (±1.6)	579	609	60	68
C	0.5 (±0.2)	4.9 (±1.6)	7.0 (±2.2)	575	606	52	70
D	0.4 (±0.1)	9.5 (+3.3; −3.2)	9.4 (±3.1)	582	613	42	76
E	-	-	2.2 (±0.3)	495	535	38	55
F	-	-	2.3 (±0.3)	489	536	52	59
G	-	-	2.9 (±0.4)	522	547	48	61
H	-	-	2.8 (±0.3)	541	570	58	63
I	-	-	3.2 (±0.5)	561	586	52	65
J	-	-	4.8 (±0.5)	598	628	17	72

**Table 6:** ICP OES data obtained from C and D.

	Atomic Content (Normalized to in Atomic Content) [a.u.]								
QD Sample	In	P	Zn	S	Se	C	H	O	ZnS: InP
C	1.00	0.61	3.60	2.71	0.66	24.43	49.20	3.20	**4.54**
D	1.00	0.61	13.31	11.92	0.32	66.18	133.67	8.94	**15.67**

**Figure A5:**
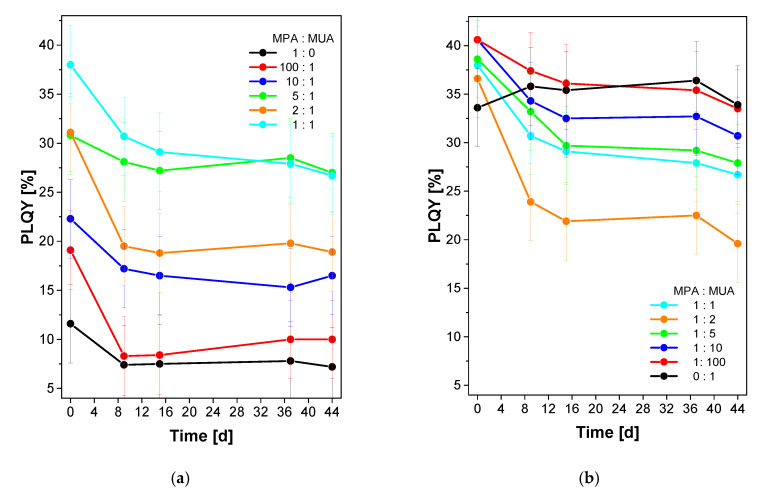
Absolute PLQY measurements of about 44 days show the long term stability of C QDs coated with different MPA:MUA ratios after dialysis storing at air and in the dark (**a**) MPA:MUA ratio from 1:0 to 1:1; (**b**) MPA:MUA ratio from 1:1 to 0:1 with corresponding error bars.

## Appendix B

**Table A1:** Interplanar spacings and corresponding Miller indices of InP and ZnS zinc blende structure obtained from ref. [45,46].

hkl	d(hkl) [Å]	
	InP Zinc Blende	ZnS Zinc Blende
(111)	3.3883	3.1231
(200)	2.9344	2.7046
(220)	2.0749	1.9125
(311)	1.7695	1.631
(222)	1.6942	1.5615

## Appendix C

**Table A2:** Interplanar spacings and corresponding Miller indices of sample E obtained from electron diffraction. The 1.7 nm spacing may be due to either one of the Miller planes indicated in the table.

d(hkl) [Å]	hkl	Structure
3.3	(111)	InP zinc blende
2.0	(220)	InP zinc blende
1.7	(222) (311)	InP zinc blende

## Appendix D

**Table A3:** Interplanar spacings and corresponding Miller indices of sample J obtained from electron diffraction. The 1.7 nm spacing may be due to either one of the Miller planes indicated in the table.

d(hkl) [Å]	hkl	Structure
3.1	(111)	ZnS zinc blende
3.4	(111)	InP zinc blende
1.9	(220)	ZnS zinc blende
2.0	(220)	InP zinc blende
1.6	(311)	ZnS zinc blende
1.7	(222) (311)	InP zinc blende

## Appendix E

**Table A4:** 1st exciton absorption wavelength and corresponding diameters of samples E to J.

Sample	1st Exciton Absorption Wavelength [nm]	Diameter [nm]
E	495	2.16 ± 0.32
F	489	2.26 ± 0.32
G	522	2.91 ± 0.41
H	541	2.76 ± 0.30
I	561	3.22 ± 0.48
J	598	4.83 ± 0.48

## Appendix G

**Table A5:** Optical properties of samples A, B, C and D coated with MPA and MUA ligands.

QD Sample Name	Ligand	PLQY [%]	Emission Peak Wavelength [nm]	FWHM [nm]
A	MPA	13	649	72
	MUA	19	649	73
B	MPA	27	619	70
	MUA	45	620	72
C	MPA	18	618	66
	MUA	44	610	71
D	MPA	24	615	78
	MUA	39	614	76

## Appendix I

**Table A6:** Optical properties of samples C and D coated with different molar ratios of MPA and MUA ligands before dialysis.

Reference Sample	MPA:MUA Ratio	PLQY [%]	Emission Peak Wavelength [nm]	FWHM [nm]
C	1:0	12	613	70
	100:1	12	618	73
	10:1	18	617	71
	5:1	20	614	72
	2:1	24	615	71
	1:1	43	610	69
	1:2	43	615	69
	1:5	44	610	70
	1:10	45	610	70
	1:100	44	608	70
	0:1	44	607	70
D	1:0	26	610	76
	100:1	13	612	79
	10:1	32	616	78
	5:1	27	610	78
	2:1	34	613	76
	1:1	36	613	75
	1:2	30	608	79
	1:5	35	612	77
	1:10	35	609	78
	1:100	34	611	77
	0:1	33	611	77

## Appendix J

**Table A7:** Optical properties of samples C and D coated with different molar ratios of MPA and MUA ligands after dialysis.

Reference Sample	MPA:MUA Ratio	PLQY [%]	Emission Peak Wavelength [nm]	FWHM [nm]
C	1:0	11	618	67
	100:1	19	618	66
	10:1	22	619	65
	5:1	31	610	68
	2:1	31	610	68
	1:1	38	614	68
	1:2	37	616	69
	1:5	39	613	70
	1:10	41	610	71
	1:100	41	608	72
	0:1	34	610	71
D	1:0	22	613	75
	100:1	27	616	75
	10:1	30	613	76
	5:1	32	611	76
	2:1	33	610	75
	1:1	33	612	75
	1:2	34	609	76
	1:5	35	610	76
	1:10	35	610	77
	1:100	33	610	77
	0:1	32	611	76
